# Severe infections requiring intensive care unit admission in patients receiving ibrutinib for hematological malignancies: a groupe de recherche respiratoire en réanimation onco-hématologique (GRRR-OH) study

**DOI:** 10.1186/s13613-023-01219-5

**Published:** 2023-12-06

**Authors:** Louise Baucher, Virginie Lemiale, Adrien Joseph, Florent Wallet, Marc Pineton de Chambrun, Alexis Ferré, Romain Lombardi, Laura Platon, Adrien Contejean, Charline Fuseau, Laure Calvet, Frédéric Pène, Achille Kouatchet, Djamel Mokart, Elie Azoulay, Antoine Lafarge

**Affiliations:** 1 Médecine Intensive Réanimation, Hôpital Saint Louis, AP-HP, Université Paris Cité, Paris, France; 2 Sorbonne Université, Paris, France; 3 Médecine Intensive Réanimation, Hospices Civils de Lyon, Lyon, France; 4 Service de Médecine Intensive-Réanimation, Sorbonne Université, Assistance Publique-Hôpitaux de Paris (APHP), Hôpital de La Pitié-Salpêtrière, Paris, France; 5 Sorbonne Université, INSERM, UMRS_1166-ICAN, Institut de Cardiométabolisme Et Nutrition (ICAN), 75013 Paris, France; 6 Réanimation Médico-Chirurgicale, Centre Hospitalier de Versailles, Le Chesnay, France; 7 Médecine Intensive Réanimation, Hôpital Pasteur, Nice, France; 8 Médecine Intensive Réanimation, Hôpital Lapeyronie, Montpellier, France; 9 Equipe Mobile d’infectiologie, Hôpital Cochin, Paris, France; 10 Hématologie, Institut de Cancérologie (ICANS), Strasbourg, France; 11 Médecine Intensive Réanimation, Hôpital Gabriel Montpied, Clermont-Ferrand, France; 12 Médecine Intensive Réanimation, Hôpital Cochin, Paris, France; 13 Médecine Intensive Réanimation, Hôpital d’Angers, Angers, France; 14 Anesthésie Réanimation, Institut Paoli Calmettes, Marseille, France

**Keywords:** Ibrutinib, Opportunistic infections, Intensive care, Lymphoproliferative disease, Targeted drugs

## Abstract

**Background:**

In the last decade, Ibrutinib has become the standard of care in the treatment of several lymphoproliferative diseases such as chronic lymphocytic leukemia (CLL) and several non-Hodgkin lymphoma. Beyond Bruton tyrosine kinase inhibition, Ibrutinib shows broad immunomodulatory effects that may promote the occurrence of infectious complications, including opportunistic infections. The infectious burden has been shown to vary by disease status, neutropenia, and prior therapy but data focusing on severe infections requiring intensive care unit (ICU) admission remain scarce. We sought to investigate features and outcomes of severe infections in a multicenter cohort of 69 patients receiving ibrutinib admitted to 10 French intensive care units (ICU) from 1 January 2015 to 31 December 2020.

**Results:**

Median time from ibrutinib initiation was 6.6 [3–18] months. Invasive fungal infections (IFI) accounted for 19% (n = 13/69) of severe infections, including 9 (69%; n = 9/13) invasive aspergillosis, 3 (23%; n = 3/13) Pneumocystis pneumonia, and 1 (8%; n = 1/13) cryptococcosis. Most common organ injury was acute respiratory failure (ARF) (71%; n = 49/69) and 41% (n = 28/69) of patients required mechanical ventilation. Twenty (29%; n = 20/69) patients died in the ICU while day-90 mortality reached 55% (n = 35/64). In comparison with survivors, decedents displayed more severe organ dysfunctions (SOFA 7 [5–11] vs. 4 [3–7], p = 0.004) and were more likely to undergo mechanical ventilation (68% vs. 31%, p = 0.010). Sixty-three ibrutinib-treated patients were matched based on age and underlying malignancy with 63 controls receiving conventional chemotherapy from an historic cohort. Despite a higher median number of prior chemotherapy lines (2 [1–2] vs. 0 [0–2]; p < 0.001) and higher rates of fungal [21% vs. 8%, p = 0.001] and viral [17% vs. 5%, p = 0.027] infections in patients receiving ibrutinib, ICU (27% vs. 38%, p = 0.254) and day-90 mortality (52% vs. 48%, p = 0.785) were similar between the two groups.

**Conclusion:**

In ibrutinib-treated patients, severe infections requiring ICU admission were associated with a dismal prognosis, mostly impacted by initial organ failures. Opportunistic agents should be systematically screened by ICU clinicians in this immunocompromised population.

## Background

Recent advancements in our understanding of carcinogenesis of hematological malignancies have paved the way for the development of targeted therapies, aiming to address the limited efficacy of conventional chemotherapy and reduce its associated toxicity. In the last decade, the emergence of targeted drugs against B-cell receptor (BCR) has reshaped the standard of care of B-cell malignancies with durable responses reported even in patients with refractory disease [1–5].

Ibrutinib, an irreversible inhibitor of Bruton tyrosine kinase (BTK), has become a key molecule in the treatment of chronic lymphocytic leukemia (CLL), mantle cell lymphoma (MCL), Waldenström macroglobulinemia (WM), diffuse large B-cell lymphoma (DLBCL), and follicular lymphoma [6–12].

BTK is a non-receptor kinase that plays a critical role in the BCR transduction pathway, driving B lymphocytes activation, differentiation, proliferation, and survival [13, 14]. Beyond BTK blockade, ibrutinib also shows broad immunomodulatory effects that have been shown to be associated with infectious complications, including opportunistic infections [15–17]. Disease status, prior chemotherapy and neutropenia have been shown to influence the incidence of severe infections [12, 18], which is one of the main reasons for ibrutinib discontinuation [19].

Because the use of targeted therapies is expanding, the need for critical care management of toxicities related to these new drugs is likely to grow. However, data focusing on most severe infections leading to organ failure remain scarce and generally extrapolated from randomized control trials [16, 20].

Accordingly, we sought to investigate features and outcomes of severe infections requiring intensive care unit (ICU) admission in a multicenter cohort of patients receiving ibrutinib for a lymphoproliferative disease, with a special focus on opportunistic infections.

## Methods

This study was approved by the institutional review board of the French Intensive Care Society. Patient’s consent was waived for this retrospective study.

### Patients

All consecutive patients receiving ibrutinib for a hematological malignancy admitted to 10 French ICU departments affiliated to the Groupe de Recherche Respiratoire en Réanimation Onco-Hématologique (GRRR-OH [21]) from 1 January 2015 to 31 December 2020 were screened for inclusion. Only patients admitted for severe infections were included in the study analysis. In case of multiple ICU admissions, only the first stay was considered.

### Data collection

Patient’s demographics, underlying disease, treatments exposure, clinical outcomes, and microbiological analysis were collected from individual medical records by three independent investigators. Primary endpoint of this study was ICU mortality, defined as death from any cause within ICU stay. Ibrutinib discontinuation and disease status evolution were also recorded.

### Definitions

Organ injuries were reported using Early Warning System definitions (oxygen requirement, low blood-pressure, altered mental status) and biological definitions used in the SOFA score [22, 23].

All types of severe infections requiring ICU admission and occurring any time from ibrutinib initiation until 3 months after its discontinuation were considered. Severe infections were identified by reviewing patient medical record, laboratory data, imaging studies and histopathological or cytology results when available. For cases with microbiological and/or radiological findings suggestive of infection, we further reviewed the clinical chart to confirm the presence of associated symptoms to exclude colonization and ascertain clinical outcome.

The source of infection was classified according to clinical and microbiological criteria following the Center for Disease Control guidelines [24]. In patients with multiple infections, only the main infectious episode leading to ICU admission was considered for the analysis. Invasive fungal infections (IFI) were defined according to the 2020 Revision and Update of the Consensus Definition of Invasive Fungal Disease from the EORTC/MSGERC Consensus Group [25]. Neutropenia was defined by a neutrophil count < 500/mm3.

Additionally, infectious features and outcomes were compared between 63 ibrutinib-treated patients and controls receiving conventional chemotherapies from an historic cohort reported by the same research network (TRIAL-OH [21]).

### Statistical analysis

Quantitative variables were described as median with interquartile range (IQR) and compared using Wilcoxon’s test. Qualitative variables were described as count and percentages and compared using Fisher’s exact test. ICU survivors and decedents were compared using univariate analysis. Due to small sample size and various underlying diseases, multivariate analysis was not relevant. Second, ibrutinib-treated patients and controls were matched using a 1:1 algorithm based on age and underlying malignancy. Ibrutinib-treated patients for WM and graft-versus-host disease were excluded from the matched analysis because these diseases were not represented in TRIAL-OH study [21]^.^ Survival functions were computed using Kaplan–Meier’s estimates on right-censored data and group comparison was performed using univariate analysis.

All tests were two-sided, and a p-value of less than 0.05 was considered significant. Statistical analyses were carried out using R version 4.2.3 (http://www.R-project.org) with the following packages: Matchit, Survival and survminer.

## Results

### Patient characteristics

**Fig. 1:**
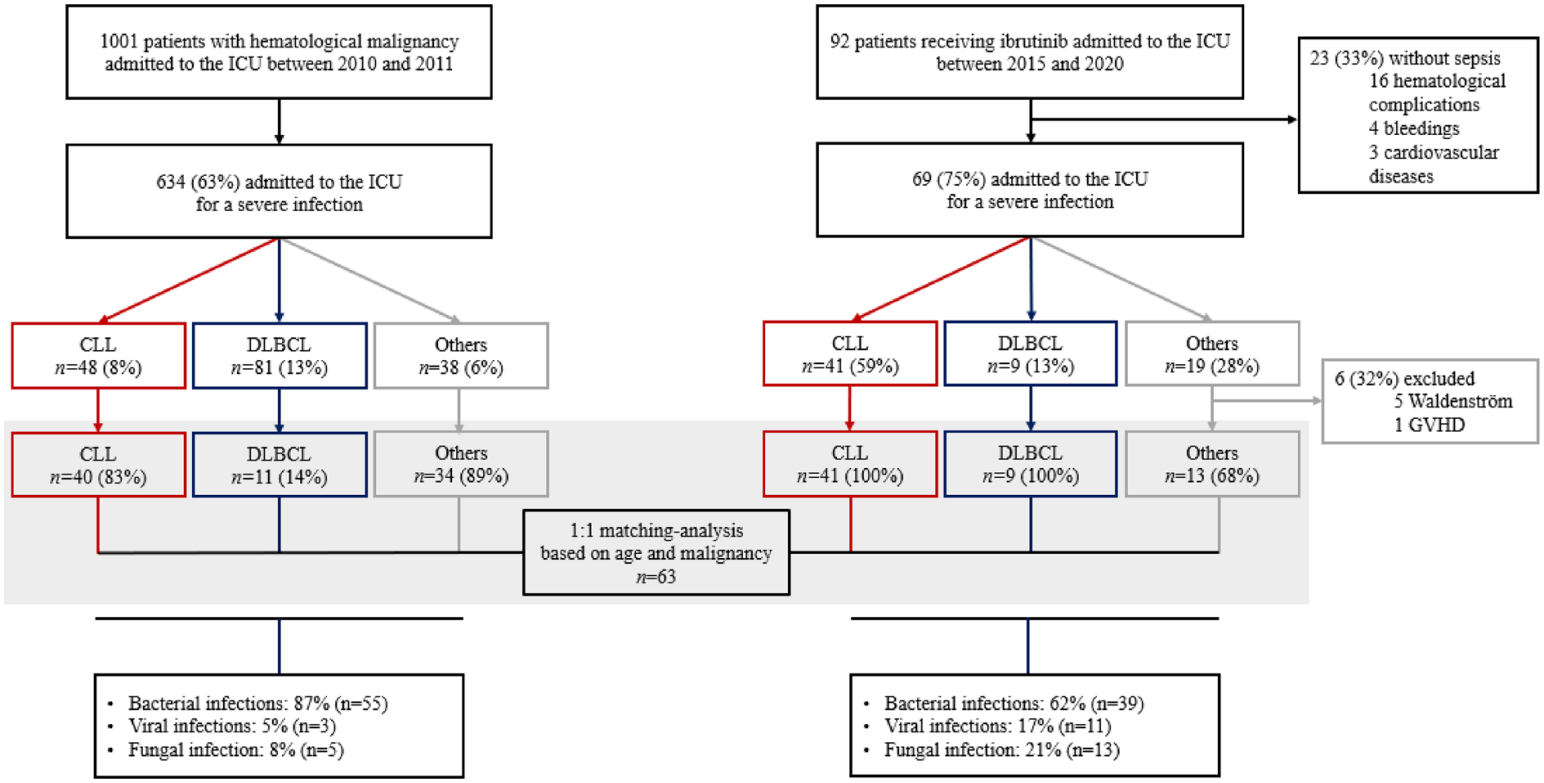
Patient’s flow diagram. Infection type and outcomes of critically ill patients receiving ibrutinib

Among 92 critically ill patients receiving ibrutinib admitted to the ICU within the study period, 75% (*n* = 69/92) were admitted for a severe infection and were included in our cohort (Fig. 1). Other adverse events included hematological complications such as tumor lysis syndrome or disease progression (*n* = 16), cardiovascular diseases (*n* = 3), and bleedings (*n* = 4).

Description of the overall study population is detailed in Table 1. Median age at ICU admission was 73 [IQR 66–78] years and 64% (*n* = 44/69) were male. The most common underlying malignancy was CLL (59%; *n* = 41/69). Other hemopathies included DLBCL (13%; *n* = 9/69), MCL (15%; *n* = 10/69), Waldenström macroglobulinemia (7%; *n* = 5/69), marginal zone lymphoma (4%; *n* = 3/69), and graft-versus-host disease (1%; *n* = 1/69). This last patient had undergone allogenic stem cell transplantation over three years before admission to the ICU.

**Table 1:** Patient characteristics and comparison between ICU survivors and decedents

	Overall (*n* = 69)	Survivors (*n* = 49)	Decedents (*n* = 20)	*P* value	Missing data
Age in years, median [IQR]	73 [66–78]	74 [67–78]	70 [61–76]	0.133	0 (0)
Male gender, *n* (%)	44 (64)	31 (63)	13 (65)	1.000	0 (0)
Prior chemotherapy lines, *n* (%)				0.467	0 (0)
0	7 (10)	4 (8)	3 (17)		
1	25 (37)	21 (43)	4 (22)		
2	20 (30)	13 (27)	7 (39)		
3	11 (16)	9 (18)	2 (11)		
≥ 4	4 (6)	2 (4)	2 (11)		
Prior chemotherapy lines, median [IQR]	2 [1–2]	1 [1–2]	2 [1–2]	0.728	0 (0)
Combination therapy, *n* (%)	23 (33)	18 (37)	5 (25)	0.342	0 (0)
Underlying disease, *n* (%)				0.218	0 (0)
CLL	41 (59)	29 (59)	12 (60)		
DLBCL	9 (13)	9 (18)	0 (0)		
Others	19 (28)	11 (22)	8 (40)		
Time between ibrutinib introduction and ICU admission in months, median [IQR]	6.6 [2.6–17.5]	8.3 [3.1–15.3]	6.2 [2.0–22.9]	0.956	2 (3)
Anti-*pneumocystis* prophylaxis, *n* (%)	40 (58)	29 (59)	11 (55)	0.960	2 (3)
Anti-viral prophylaxis, *n* (%)	45 (65)	34 (69)	11 (55)	0.390	2 (3)
Other comorbidities and additional infectious risk factors, *n* (%)					0 (0)
Cardiovascular disease	41 (59)	31 (63)	10 (50)	0.323	
Chronic respiratory disease	12 (17)	8 (16)	4 (20)	0.691	
Chronic kidney disease	5 (7)	3 (6)	2 (10)	0.562	
Chronic liver disease	5 (7)	4 (8)	1 (5)	0.663	
Hypogammaglobulinemia	16 (23)	13 (27)	3 (15)	0.290	
Neutropenia	7 (10)	6 (12)	1 (5)	0.382	
Diabetes	13 (19)	8 (16)	5 (25)	0.619	
HIV	1 (1)	0 (0)	1 (5)	0.641	
SOFA score at ICU admission, median [IQR]	5 [3–8]	4 [3–7]	7 [5–11]	0.004	2 (3)
Organ injuries at ICU admission, *n* (%)					
Respiratory	49 (71)	30 (61)	19 (95)	0.005	0 (0)
Hemodynamic	34 (49)	22 (45)	12 (60)	0.262	0 (0)
Liver	13 (19)	11 (22)	2 (10)	0.248	2 (3)
Renal	23 (33)	14 (29)	9 (45)	0.205	0 (0)
Neurological	30 (43)	18 (37)	12 (60)	0.083	0 (0)
Multiorgan failure	47 (68)	31 (63)	16 (80)	0.173	2 (3)
Diagnostic group, *n* (%)				0.520	0 (0)
Bacterial infection	45 (65)	34 (69)	11 (55)		
Fungal infection	13 (19)	8 (16)	5 (25)		
Viral infection	11 (16)	7 (14)	4 (20)		
Polymicrobial infections	12 (17)	8 (16)	4 (20)	0,715	
Organ support throughout ICU stay, *n* (%)					
Vasopressors	39 (57)	24 (49)	15 (75)	0.087	0 (0)
IMV	28 (41)	15 (31)	13 (68)	0.010	0 (0)
RRT	10 (15)	5 (10)	5 (25)	0.227	0 (0)
ICU length of stay in days, median [IQR]	5 [2–11]	6 [3–12]	3 [2–8]	0.105	0 (0)
Ibrutinib discontinuation in the ICU	57 (83)	41 (84)	16 (80)	0.691	0 (0)
Outcomes, *n* (%)					
ICU mortality	20 (29)	–	–		0 (0)
Hospital mortality	32 (50)	12 (27)	–		5 (7)
Day-90 mortality	35 (55)	15 (34)	–		5 (7)

CLL: Chronic Lymphocytic Leukemia, DLBCL: Diffuse Large B-Cell Lymphoma, ICU: Intensive Care Unit, HIV: Human Immunodeficiency Virus, IMV: Invasive Mechanical Ventilation, RRT: Renal Replacement Therapy, IQR: Interquartile Range

Median time from ibrutinib initiation to ICU admission was 6.6 months [IQR 2.6–17.5]. Median number of prior therapy lines was 2 [1, 2], including 29 (42%; *n* = 29/69) patients treated by other chemotherapy within the last 6 months, and 7 (10%; *n* = 7/69) patients receiving ibrutinib as first line therapy. Prior therapy lines included anti-CD20 therapy, fludarabine, and alemtuzumab in 54 (78%; *n* = 54/69), 24 (35%; n = 24/69) and 2 (3%; *n* = 2/69) patients respectively. Most patients (67%; *n* = 46/69) received ibrutinib as monotherapy. In patients on combination therapy (33%; *n* = 23/69), concurrent treatments included anti-CD20 monoclonal antibodies in 48% (*n* = 11/23) and corticosteroids in 39% (*n* = 9/23). Additional infectious risk factors included diabetes in 19% (*n* = 13/69), hypogammaglobulinemia in 23% (*n* = 16/69), neutropenia in 10% (*n* = 7/69), and HIV infection in 1% (*n* = 1/69) of patients. Anti-*Pneumocystis* and anti*-*viral prophylaxis were administered to 58% (*n* = 40/69) and 65% (*n* = 45/69) of patients respectively.

### Types of infection

Among 69 infectious episodes leading to ICU admission, the most frequent were bacterial infections (65%; *n* = 45/69 episodes). Main bacterial infection sources were lungs (40%; *n* = 18/45) and urinary tract (16%; *n* = 7/45), while 6 (13%) catheter-related infections were detected.

Regarding viral infections, 11 (16%) episodes were recorded. Severe acute coronavirus 2 (SARS-CoV-2) infections accounted for 54% (*n* = 6/11) of viral infections. Other viral episodes included cytomegalovirus infections in 3 (27%; *n* = 3/11) patients, herpes simplex virus reactivation and disseminated adenovirus infection in 1 patient each.

Overall, 13 (19%) fungal episodes were reported in the whole cohort (Table 2), including 11 (84%; *n* = 11/13) patients with CLL, 3 (23%; *n* = 3/13) patients receiving concomitant corticosteroids therapy and 1 (8%; *n* = 1/13) allogeneic hematopoietic stem-cell recipient. The proportion of IFI was not significantly higher in CLL patients in comparison with other hematological malignancies (27% vs. 11%, *p* = 0.105). Additional risk factors included neutropenia in 8% (*n* = 1/13), hypogammaglobulinemia in 15% (*n* = 2/13) and diabetes in 23% (*n* = 3/13). The most frequent fungal infection was proven or probable invasive aspergillosis (69%; *n* = 9/13). Other fungal episodes included *Pneumocystis jirovecii* pneumonia in 3 (23%; *n* = 3/13) patients and *Cryptococcus neoformans* infection in 1 (8%; *n* = 1/13) patient.

**Table 2:** Characteristics and outcomes of critically ill patients receiving ibrutinib with invasive fungal infection

	Underlying malignancy and treatment line	Concomitant treatments and additional risk factors	Organ failure	Organ support	EORTC grade	Organ involvement	Anti-infective treatment	ICU outcome	Day-90 outcome
Aspergillosis									
1	CLL Second line	–	ARF	–	Probable	Lungs	VCZ	Death	Death
2	CLL Fourth line	CTC Diabetes	ARF ALF	IMV NAD	Probable	Lungs	VCZ AmB	Alive	Alive
3	CLL Second line	HypoƔ	ARF Shock AKI	IMV NAD RRT	Probable	Lungs	VCZ	Alive	Alive
4	CLL Second line	–	ARF	IMV NAD	Probable	Lungs	TMP-SMX VCZ	Alive	Alive
5	CLL First line	Diabetes	ARF Shock	IMV NAD RRT	Probable	Lungs	VCZ	Death	Death
6	CLL Third line	–	ARF	-	Probable	Lungs	VCZ	Alive	Death
7	CLL Third line	CTC	ARF	IMV NAD RRT	Probable	Lungs CNS	VCZ Caspo	Death	Death
8	CLL Second line	–	ARF Shock AKI	IMV NAD	Probable	Lungs	Caspo AmB	Death	Death
9	CLL Third line	Neutropenia Allo-HSCT	ARF Shock Coma	IMV NAD	Probable	Lungs	AmB	Death	Death
*Pneumocystis* pneumonia									
1	CLL Second line	HypoƔ	ARF AKI	–	Probable	Lungs	TMP-SMX	Alive	Alive
2	DLBCL Second line	CTC Anti-CD20	ARF Shock Coma	IMV NAD	Probable	Lungs	TMP-SMX	Alive	Death
3	DLBCL First line	Diabetes Anti-CD20	ARF Shock	IMV NAD	Probable	Lungs	TMP-SMX	Alive	Alive
Cryptococcosis									
1	CLL Fourth line	–	ARF ALF AKI	IMV RRT	Proven	Lungs	AmB 5FU FLZ	Alive	Death

AKI: Acute Kidney Injury, ALF: Acute Liver Failure, Allo-HSCT: Allogeneic Hematopoietic Stem-Cell Transplantation, AmB: Amphotericin B, Anti-CD20: Anti-CD20 monoclonal antibodies, ARF: Acute respiratory failure, Caspo: Caspofungin, CLL: Chronic Lymphocytic Leukemia, CNS: Central Nervous System, CTC: Corticosteroid, DLBCL: Diffuse Large B-Cell Lymphoma, FLZ: Fluconazole, HypoƔ: Hypogammaglobulinemia, ICU: Intensive Care Unit, IMV: Invasive Mechanical Ventilation, NAD: Vasopressors, RRT: Renal Replacement Therapy, TMP-SMX: Cotrimoxazole, VCZ: Voriconazole, 5FU: Flucytosine

The spectrum of infections was similar between patients receiving ibrutinib as single-drug therapy and patients on combination regimen, including 21% (*n* = 10/46) and 13% (*n* = 3/23) of fungal episodes respectively (*p* = 0.422).

Overall, 81 infections were reported among the 69 patients, including 54 bacterial infections (67%; *n* = 54/81), 14 viral infections (17%; *n* = 14/81) and 13 fungal infections (16%; *n* = 13/81). Twelve (17%; *n* = 12/69) patients displayed polymicrobial infections, including 8 (12%; *n* = 8/69) patients with IFI associated with bacterial (75%; *n* = 6/8) or viral coinfections (25%; *n* = 2/8).

### ICU admission

At ICU admission, most patients (68%; *n* = 47/69) displayed multi-organ failure and median SOFA score was 5 [IQR 3–8]. Most common organ injury at admission was acute respiratory failure (ARF) (71%; *n* = 49/69), including all patients with IFI. Overall, invasive mechanical ventilation (IMV) was required in 41% (*n* = 28/69) of patients. The median time from ICU admission to IMV was inferior to one day [IQR 0–1]. At ICU admission, shock, and acute kidney injury (AKI) were reported in 49% (*n* = 34/69) and 33% (*n* = 23/69) patients respectively. Renal replacement therapy (RRT) was initiated in 15% (*n* = 10/69) patients throughout the ICU stay. Median time from hospital to ICU admission was 2 [IQR 1–13] days.

### Outcomes and predictive factors of ICU mortality

Median ICU and hospital lengths of stay were 5 [2–11] and 21 [IQR 7–43] days respectively. Twenty (29%; *n* = 20/69) patients died in the ICU. End-of-life decisions preceded ICU deaths in 65% (*n* = 13/20). Comparison of baseline characteristics between ICU survivors and decedents is summarized in Table 1. There was no difference regarding baseline characteristics, underlying malignancy, and infection type. Of note, the proportion of polymicrobial infections was similar between ICU survivors and decedents (20 vs. 16%; *p* = 0.715). Organ dysfunctions at ICU admission were more severe in non-survivors (SOFA 7 [IQR 5–11] vs. 4 [IQR 3–7], *p* = 0.004. In comparison with survivors, decedents were more likely to display ARF at ICU admission (95% vs. 61%, *p* = 0.005) and to require IMV (68% vs. 31%, *p* = 0.010).

**Fig. 2:**
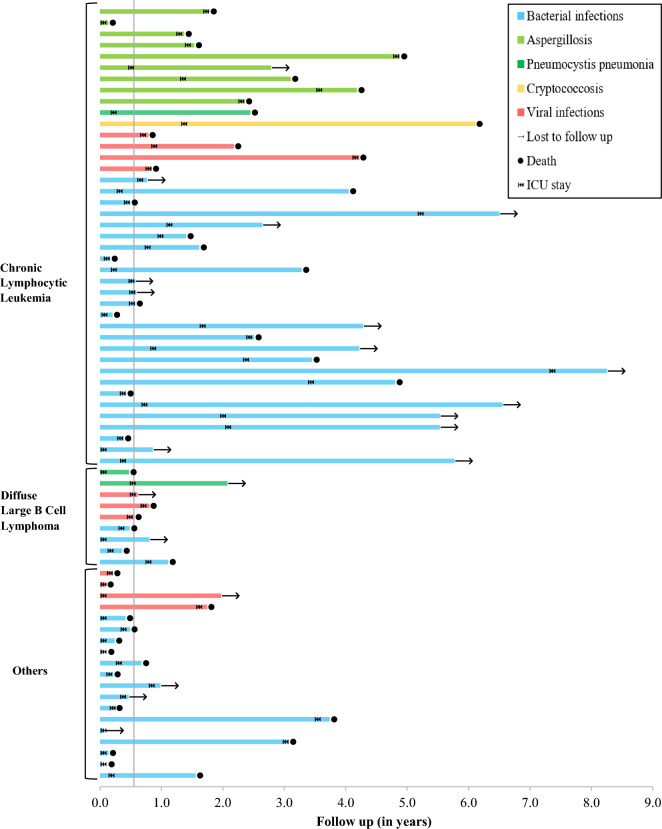
Swimmer’s plot of patients admitted to the ICU for severe infections, from ibrutinib introduction to loss to follow up or death. Patients are classified by underlying disease group and final diagnosis during ICU stay. The straight line represents the median time from ibrutinib introduction to ICU admission

Follow up data after ICU discharge was missing for 5 patients. Overall, hospital mortality reached 50% (*n* = 32/64) and day-90 mortality 55% (*n* = 35/64), affecting 34% (*n* = 15/44) of ICU survivors. Ibrutinib was discontinued during the ICU stay in 57 patients (83%; *n* = 57/69), including all patients with IFI. Among survivors in whom ibrutinib treatment had been discontinued through ICU stay, discontinuation was permanent in 65% (*n* = 24/37) and only temporary in 35% (*n* = 13/37). After a median follow up of 5 [IQR 1–18] months, 61% (*n* = 27/44) of ICU survivors displayed B-cell malignancy progression or recurrence. Individual patient clinical trajectories from ibrutinib introduction to loss of follow up or death are displayed in Fig. 2.

### Comparison between ibrutinib-treated patients and controls receiving conventional chemotherapy

Severe infections features and outcomes were compared between 63 ibrutinib-treated patients and 63 controls receiving conventional chemotherapy from an historic cohort (TRIAL-OH (21)), matched on age and underlying malignancy (Table 3). Median number of prior chemotherapy lines was higher in ibrutinib-treated patients in comparison with controls (2 [IQR 1–2] vs. 0 [0–2] p = 0.001). Other baseline characteristics were similar between the 2 groups.

**Table 3:** Matched comparison of ibrutinib-treated patients and controls receiving conventional chemotherapies

	Ibrutinib-treated patients (*n* = 63)	Control patients (*n* = 63)	*P* value
Age in years, median [IQR]	74 [66–78]	72 [66–78]	0.895
Male gender, *n* (%)	40 (63)	42 (66)	0.852
Prior chemotherapy lines, *n* (%)			< 0.001
0	7 (12)	15 (24)	
1	22 (36)	23 (37)	
2	18 (30)	6 (10)	
3	11 (18)	12 (19)	
≥ 4	3 (4)	6 (9)	
Prior chemotherapy lines, median [IQR]	2 [1–2]	0 [0–2]	0.001
Underlying disease, *n* (%)			0.881
CLL	41 (65)	40 (64)	
DLBCL	9 (14)	11 (18)	
Others	13 (21)	12 (19)	
Additional risk factors, *n* (%)			
Diabetes	11 (18)	12 (19)	1.000
HIV	1 (2)	1 (2)	1.000
SOFA score at ICU admission, median [IQR]	5 [3–8]	7 [5–10]	0.065
Organ injury at ICU admission, *n* (%)			
Respiratory	45 (71)	49 (79)	0.401
Hemodynamic	32 (50)	33 (52)	0.858
Liver	12 (19)	0 (0)	< 0.001
Renal	21 (33)	19 (30)	0.710
Neurological	27 (43)	12 (19)	0.004
Multiorgan failure	43 (68)	40 (64)	0.571
Organ support throughout ICU stay, *n* (%)			
Invasive mechanical ventilation	26 (41)	39 (62)	0.032
Vasopressors	35 (56)	44 (70)	0.141
RRT	9 (14)	5 (8)	0.395
Diagnostic group, *n* (%)			0.004
Bacterial infection	39 (62)	55 (87)	0.039
Viral infection	11 (17)	3 (5)	0.027
Fungal infection	13 (21)	5 (8)	0.001
ICU mortality, *n* (%)	17 (27)	24 (38)	0.254
Day-90 mortality, *n* (%)	30 (52)	29 (48)	0.785

CLL: Chronic Lymphocytic Leukemia, DLBCL: Diffuse Large B-Cell Lymphoma, ICU: Intensive Care Unit, HIV: Human Immunodeficiency Virus, RRT: Renal Replacement Therapy, IQR: Interquartile Range

Regarding infection type, ibrutinib-treated patients were more likely to display IFI (21% [*n* = 13/63] vs. 8% [*n* = 5/63], *p* = 0.001) or viral episodes (17% [*n* = 11/63] vs. 5% [*n* = 3/63], *p* = 0.027) while infections in patients receiving conventional chemotherapy were more frequently bacterial (87% [*n* = 55/63] vs. 62% [*n* = 39/63], *p* = 0.039).

Median SOFA score at ICU admission was similar between the 2 groups but ibrutinib-treated patients were more likely to display acute liver injury (19% [*n* = 12/63] vs. 0% [*n* = 0/63], *p* < 0.001) and impaired consciousness (43% [*n* = 27/63] vs. 19% [*n* = 12/63], *p* = 0.004). There was no difference regarding ARF incidence, but control patients were more likely to undergo IMV (62% [*n* = 39/63] vs. 41% [*n* = 26/63], *p* = 0.032).

**Fig. 3:**
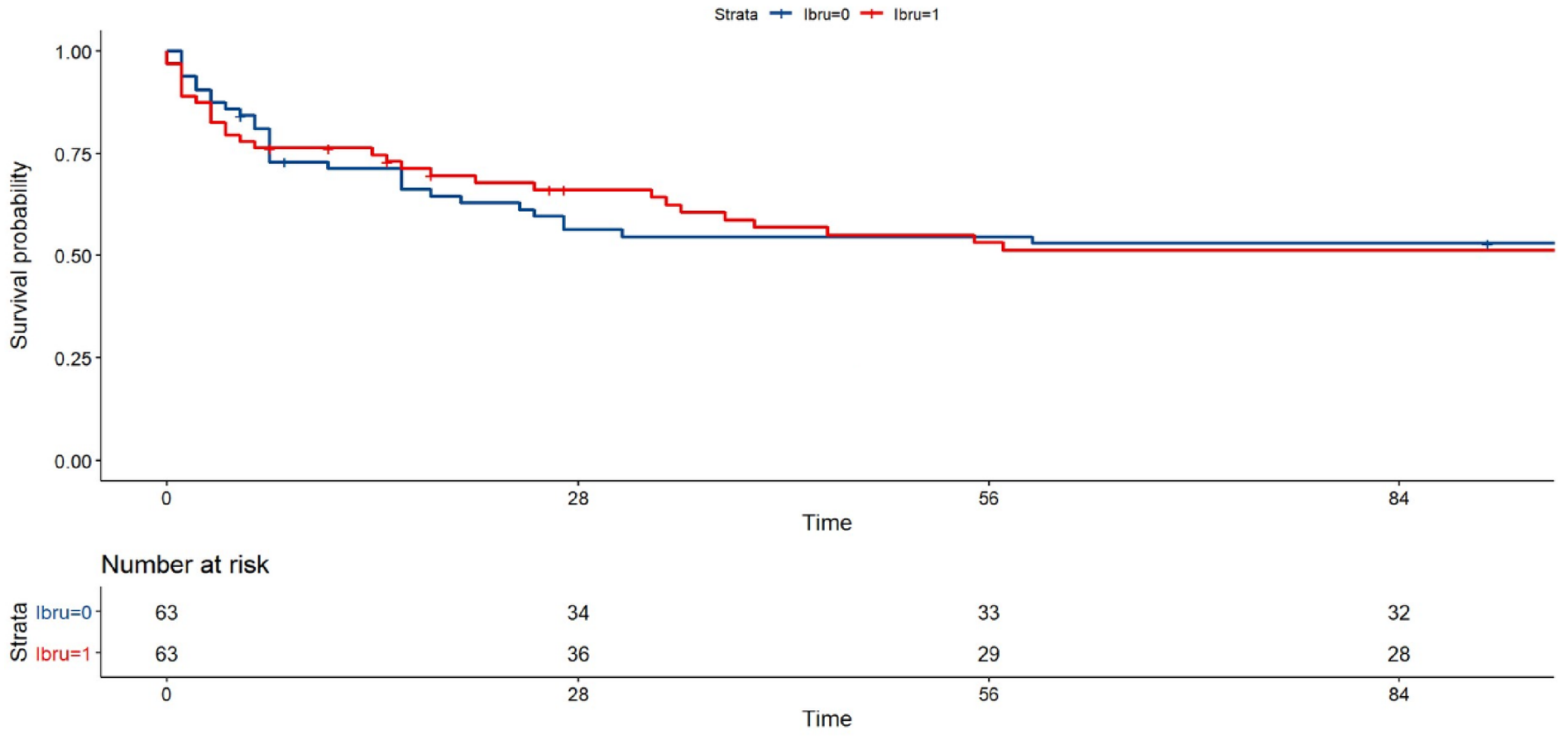
Survival curves of ibrutinib-treated patients and controls receiving conventional chemotherapy. Follow-up in months

However, ibrutinib-treated patients and controls receiving conventional chemotherapies displayed similar ICU (27% vs. 38%, *p* = 0.254) and day-90 (52% vs. 48%, *p* = 0.785) mortality rates (Fig. 3).

## Discussion

This is the first series focusing on severe infectious episodes requiring ICU admission in patients receiving ibrutinib in a real-life setting. Beyond the predominance of bacterial infections, our results underline the risk of opportunistic infections within the first year of treatment. Overall, severe infections requiring ICU admission are associated with a dismal prognosis, with over 50% of mortality at day 90, mostly impacted by initial organ dysfunctions.

In our cohort, infectious features corroborated recent studies not restricted to critically ill patients [16, 20]. The predominance of bacterial episodes, which accounted for nearly 60% of severe infections, was previously reported in studies focusing on ibrutinib-associated adverse events [18]. This finding may be promoted by ibrutinib off-target effects including innate immunity impairment (involving macrophages, neutrophils [26–28] and natural killer cells [29] function, and modulation of T-cell activation through Interleukin 2 Receptor Kinase (ITK) inhibition [30]. In contrast, minimal off-target effects among other members of the TEC tyrosine kinase family are reported with more specific second-generation BTK inhibitor such as acalabrutinib [30]. Further studies are warranted to determine infections incidence and severity associated with these new molecules [31, 32].

A specific concern regarding IFI in patients treated with targeted therapies including ibrutinib has previously been pointed out [12, 17, 20, 33]. In the present series, one out of five infection was an IFI. IFI proportion even reached 27% in patients admitted for acute respiratory failure, suggesting that opportunistic agents should be systematically screened by ICU clinicians (serum galactomannan, ß-D-Glucan, PCR, etc.…) in this setting. As expected, the spectrum of fungal episodes included a majority of pulmonary aspergillosis, three *Pneumocystis* pneumonia and one pulmonary cryptococcosis [17, 18, 33, 34]. Aspergillus susceptibility in ibrutinib-treated patients may rely on the critical role of BTK activation in macrophages for TNFα response, neutrophil recruitment and functions including reactive oxygen species production*,* and *A.fumigatus* clearance in the respiratory airways [26, 27, 35]. In contrast with previous reports, we report only one case of extra-pulmonary fungal dissemination in the present cohort [33, 36]. Interestingly, almost all fungal episodes were diagnosed in CLL patients with additional risk factors, including diabetes, prior chemotherapies, concomitant corticosteroids and/or anti-CD20 monoclonal antibodies. IFI proportion tended to be higher in CLL patients in comparison with other hematological malignancies in line with the data reported by Gold et al. [37].

Main limitations of this study include its retrospective design and numerous confounding factors, particularly regarding immunosuppression. Ibrutinib-related infectious burden probably partly reflects the impact of previous chemotherapies and uncontrolled malignancy status at the beginning of the targeted-drug treatment [38]. In the present cohort, the short time from ibrutinib introduction to severe infection requiring ICU admission, consistent with previous studies, supports this assumption. Infections incidence has previously been shown to be higher in the first year of ibrutinib exposure and to progressively decrease over time [2, 18, 37]. Moreover, in vitro studies have identified a partial recovery of both humoral immune function [39] and T Cell Receptor (TCR) repertoire diversity via ITK inhibition [30, 40, 41]. In line with these findings, the matched comparison between ibrutinib-treated patients and controls receiving conventional chemotherapies should not be interpretated as an attempt to differentiate molecules-related infectious adverse events. Thus, higher proportion of fungal infections may simply reflect the higher number of prior lines of chemotherapy in patients receiving ibrutinib. Given the discrepancies between the two cohorts regarding inclusion periods, baseline characteristics, organ injuries at ICU admission and IMV requirement, outcomes must be compared cautiously.

Furthermore, increased proportion of viral episodes in ibrutinib-treated patients in the present study may be due to the emergence of Severe Acute Respiratory Virus Coronavirus 2 (SARS-CoV-2) in 2020, which accounted for over half of viral infections. Several studies have shown a severe prognosis among CLL patients diagnosed with SARS CoV2 infection [42]. In a multicentre retrospective study including 198 patients with symptomatic SARS CoV-2 infection, the authors reported a mortality reaching 33% [43]. The impact of ibrutinib treatment on the course of SARS-CoV-2 infection is still uncertain, and ibrutinib discontinuation in SARS-CoV-2-infected patients remains controversial [42, 44, 45]. In our cohort only one in five SARS CoV-2 infected patients died in the ICU. However, hence the dismal prognosis among CLL patients, evaluation of ibrutinib treatment impact on the course of Coronavirus Induce Disease 2019 (COVID 19) in these patients must be taken carefully.

In this study, severe infections in critically ill patients receiving ibrutinib were associated with an unexpectedly high day-90 mortality above 50%. However, outcomes comparison with studies in which most of severe infections do not require ICU admission would be inappropriate. Organ failures at ICU admission were the major determinant of mortality, suggesting that ICU transfer should be considered as soon as these high-risk patients show any sign of organ injury. Last, severe infection conveyed the need for ibrutinib discontinuation in a high proportion of cases, but further studies are warranted to evaluate its impact on long-term outcomes.

## Conclusion

In patients receiving ibrutinib for hematological diseases, severe infections requiring ICU admission are associated with a dismal prognosis, mostly impacted by initial organ failures. Due to the high proportion of fungal infections, opportunistic agents should be systematically screened by ICU clinicians in this immunocompromised population, especially in the setting of acute respiratory failure.

## Data Availability

The datasets used and analyzed in the current study are available from the corresponding author on reasonable request.

## Abbreviations

BCR: B cell receptor
BTK: Bruton tyrosine kinase
CLL: Chronic lymphocytic leukemia
MCL: Mantel cell lymphoma
WM: Waldenström macroglobulinemia
DLBCL: Diffuse large B cell lymphoma
ICU: Intensive care unit
IFI: Invasive fungal infection
IQR: Interquartile range
HIV: Human immunodeficiency virus
ARF: Acute respiratory failure
IMV: Invasive mechanical ventilation
AKI: Acute kidney infection
RRT: Renal replacement therapy
ITK: Interleukin 2 receptor kinase
TCR: T cell receptor
